# Nutrition and non-nutrition determinants of maternal mental disorders, stress and depression in pregnancy amidst a global pandemic: findings from Action against Stunting Hub Indonesia

**DOI:** 10.1017/S0007114525105515

**Published:** 2026-03-28

**Authors:** Umi Fahmida, Min Kyaw Htet, Amanda Safiera Ameline, Tiffany Cornelia Angelin, Risatianti Kolopaking, Hilary Davies-Kershaw, Elaine L. Ferguson

**Affiliations:** 1 Department of Nutrition Science, Faculty of Medicine, Universitas Indonesia − Dr. Cipto Mangunkusumo General Hospital, Jakarta, Indonesia; 2 Southeast Asian Ministers of Education Organization Regional Centre for Food and Nutrition (SEAMEO RECFON), Pusat Kajian Gizi Regional Universitas Indonesia, Jakarta, Indonesia; 3 Sydney School of Public Health, Faculty of Medicine and Health, The University of Sydney, Sydney, NSW, Australia; 4 Sinergi Qalbu Fikri, Depok, Indonesia; 5 Faculty of Psychology, UIN Syarif Hidayatullah, Jakarta, Indonesia; 6 Department of Population Health, London School of Hygiene and Tropical Medicine Faculty of Epidemiology and Public Health, London, UK

**Keywords:** Maternal stress, iron deficiency, Common mental disorders, Maternal depression, Pregnancy

## Abstract

Pregnant women are particularly vulnerable to stress and depression, which can negatively impact birth outcomes and maternal care practices. This study aimed to investigate the prevalence and determinants of stress, depression and common mental disorders among pregnant women in East Lombok, Indonesia, during the COVID-19 pandemic. This cross-sectional study was part of the Action Against Stunting Hub. Data on maternal characteristics, dietary intake, anthropometry and biochemical status were collected. Mental health was assessed during the second and third trimesters using validated instruments, the Perceived Stress Scale, Edinburgh Postnatal Depression Scale and Self-Reporting Questionnaire. The prevalence of maternal stress, depression and common mental disorder (CMD) was 86·3 %, 26·5 % and 29·7 %, respectively. CMD was associated with iron deficiency (aOR 1·61) and not receiving government assistance (aOR 1·48). Low adherence to a healthy and diverse diet, i.e. limited intake of grains, tubers, eggs, fruits and vegetables, was associated with increased odds of antenatal stress (aOR 1·59) and CMD (aOR 1·60). For depression, significant factors included higher maternal education (aOR 2·27), low-to-moderate social support (aOR 1·72) and adherence to an unhealthy dietary pattern characterised by high sugar and fat intake (aOR 1·47). Targeted nutrition interventions, including food-based dietary recommendations and social safety net programs, are essential during pregnancy to support nutrient intake. In addition to addressing iron deficiency, integrated approaches that promote dietary diversity, provide government support to low-income households and strengthen social support networks are recommended to improve maternal mental health outcomes.

Pregnancy is a period marked by significant biological, psychological and behavioural changes that increase women’s vulnerability to a range of mental health disorders, including stress, anxiety and depression^(1,2)^. However, the causes of stress, individual responses to it and coping mechanisms vary greatly among women. Those who are undernourished or of low socio-economic status are particularly at risk for developing mental health conditions compared with their more advantaged counterparts^(3,4)^. Additionally, factors such as exposure to trauma, fear of childbirth and lack of social support can further exacerbate stress levels during pregnancy^(2,5)^.

Maternal mental disorders have been linked to a range of negative outcomes in offspring, including developmental delays, impaired attachment and difficulties with social interaction^(6)^. Despite the significant burden of these disorders, mental health conditions remain under-reported and undertreated in low- and middle-income countries^(7)^. Antenatal stress and depression are associated with an increased risk of adverse birth outcomes, including miscarriage, preterm birth, low birth weight and cesarean delivery^(8)^. Moreover, women experiencing higher levels of perceived stress are more likely to engage in poor dietary practices and unhealthy eating behaviours, such as meal skipping, emotional eating or excessive caloric intake^(5)^.

The Coronavirus Disease 2019 (COVID-19), which first emerged in Indonesia in March 2020, disrupted many aspects of life, including food systems and healthcare services. Physical distancing and mandatory lockdown limited the access of pregnant women to social support^(9)^. Social support is a multidimensional concept that includes emotional (e.g. encouragement, compassion and reassurance), instrumental (e.g. assistance with household chores, caregiving) and and informational (e.g. information exchange, problem-solving) support, and it has been considered a key indicator of women’s physical and mental well-being during pregnancy and after childbirth^(10)^. Maintaining good mental well-being throughout pregnancy can help lower the risk of adverse outcomes such as shorter gestational age, preterm birth, preeclampsia and neonatal morbidity, as maternal stress can otherwise compromise both maternal and fetal health and nutrition, increasing the likelihood of these complications^(11)^. In addition to the pandemic, limited local job opportunities often require men to work away from their home regions. As a result, many were unable to return due to travel restrictions, leaving pregnant women feeling more isolated and unsupported during this period^(12)^.

Evidence suggests that consuming a healthy, high-quality diet can improve mental health and mood^(13,14)^. During pregnancy, adherence to a healthy dietary pattern, characterised by high intake of pulses, nuts, vegetables, fruits, fish, shellfish, seafood products and dairy, has been associated with a decreased risk of depression^(13)^. This also indicated increasing intake of key nutrients such as B vitamins (B_1_, B_2_, B_6_, B_12_ and folate) to help protect against depression^(13)^. In addition, vitamin D, zinc (Zn), magnesium (Mg), selenium (Se), iron (Fe), calcium (Ca) and *n*-3 fatty acids have a significant effect on the brain and nervous system, which might influence the appearance of depressive symptoms^(15)^.

A linear programming analysis by Fahmida *et al.* (2022), conducted as part of the National Monitoring of Nutrient Consumption (*Pemantauan Konsumsi Gizi*) across ten stunting-prioritised districts in Indonesia, identified Fe, folate and Ca as key problem nutrients among pregnant women^(16)^. This finding aligns with the high prevalence of anaemia during pregnancy in the region^(17)^. Similarly, a recent systematic review found that energy and macronutrient intakes were generally below the recommended dietary allowance, with most pregnant women did not meet the estimated average requirements for vitamin D (< 70 %), vitamin E (< 50 %), water-soluble vitamins (< 80 %), as well as Ca, potassium and Fe (< 60 %)^(18)^. However, adequate intakes of vitamin A, Zn and phosphorus were observed. Regarding dietary pattern, the Rhea Birth Cohort study in Greece (2007–2009) found that women adhering to a ‘health-conscious’ dietary pattern, rich in vegetables, fruits, legumes, nuts, dairy, fish and olive oil, had significantly lower Edinburgh Postnatal Depression Scale (EPDS) scores (highest v. lowest tertile: *β*-coefficient = −1·75, *P* = 0·02)^(19)^.

Fe status is critically important during pregnancy due to physiological changes such as haemodilution, which can influence maternal health outcomes^(20)^. A previous study conducted among mothers in the Gaza Strip demonstrated an increased risk of postpartum depression in women with iron deficiency^(21)^. A cross-sectional study analysing data from the USA National Health and Nutrition Examination Survey 2005–2010 found that somatic symptoms of depression were more prevalent among nonpregnant women of reproductive age (20–44 years) with iron deficiency, indicated by elevated transferrin receptor levels, particularly those with low income^(22)^.

Limited research has been conducted in Indonesia examining the impact of maternal nutrition (both dietary intake and nutritional status) on mental health outcomes. Findings from the Indonesia Basic Health Research (2018) indicated that among pregnant women, common mental disorders (CMD) were significantly associated with first-trimester pregnancy, a history of pregnancy complications and low mid-upper arm circumference. In postpartum mothers, significant factors associated with CMD included rural residence, unwanted pregnancy, history of abortion, spontaneous delivery, pregnancy and postpartum complications, inadequate antenatal care and contraceptive use^(23)^. This study aims to investigate the association between determinants, including socio-demographic, anaemia, iron deficiency, pregnancy-related and dietary-related factors, with mental health outcomes (i.e. maternal stress, depression and common mental disorders) during pregnancy.

## Methods

### Study design and population

This cross-sectional study is a part of the observational cohort study in Indonesia entitled Action Against Stunting Hub, conducted in four rural sub-districts of East Lombok (2019–2024). The Action Against Stunting Hub study investigates interdisciplinary determinants of maternal and child nutrition, health and well-being using a ‘whole child’ approach. It conducts longitudinal assessments across pregnancy, lactation and the complementary feeding period to identify key factors influencing child growth and development. These assessments include (1) anthropometry, (2) biomarkers of nutritional and health status, (3) dietary intake, (4) fetal growth and development, (5) infant morbidity, (6) infant and young child feeding practices and (7) maternal stress, depression and social support during the perinatal period^(24)^. East Lombok district was purposively selected as the study area due to its high prevalence of stunting (above 30 %)^(17)^. East Lombok is one of the largest and most densely populated districts in the province, with an estimated total population of 1,200,612 in 2019^(25)^. The majority of inhabitants work in agriculture, fisheries and some are involved in trading sectors^(25)^. Of the twenty-one sub-districts, Aikmel, Lenek, Sikur and Sakra sub-districts were selected as our study area due to their high stunting prevalence (above 30 %)^(17)^, representation of different geographic typology, e.g. mountainous and coastal regions, and access to the laboratory within 2 hours from the time of biological sample collection.

Pregnant women were recruited between February and September 2021. Eligible participants were 18–40 years old, at 16–20 weeks of gestation, of Sasak ethnicity, planning to reside in East Lombok district for the following 30 months and provided informed consent to participate. Data collection was done in the second (T2) and third (T3) trimester of pregnancy from February 2021 to January 2022.

### Data collection

This study reports findings on maternal nutritional status and mental health based on data collected during the second and third trimesters of pregnancy. Pregnant women were recruited during their second trimester. We collected baseline characteristics and nutritional status of mothers during the second trimester, including socio-demographic and pregnancy characteristics. Subsequently, during the follow-up period (third trimester), pregnant women were interviewed using questionnaires to investigate the mental health status of the mothers EPDS, Global Measure of Perceived Stress (GMPS), Multidimensional Scale of Perceived Social Support (MSPSS) and Self-Reporting Questionnaire (SRQ) and had blood samples collected. Enumerators fluent in the local Sasak language, each with an educational background of at least a diploma in nutrition, were recruited and trained for electronic data capture using a tablet-based app (www.commcarehq.org). Household socio-demographic information, maternal and paternal characteristics were collected using standard questionnaires from previous studies and national surveys, including the National Socioeconomic Survey (SUSENAS)^(26)^, Indonesian Basic Health Survey (RISKESDAS)^(17)^ and the cohort study in East Java, Indonesia (BADUTA project)^(27)^.

### Socio-demographic and pregnancy-related characteristics

Socio-demographic characteristics included (i) age of pregnant women and husband, (ii) education level, (iii) employment status, (iv) family size, (v) tobacco smoke exposure and (vi) wealth index. Exposure to tobacco smoke was assessed based on whether women were actively smoking during pregnancy or were exposed to environmental tobacco smoke as passive smokers. The wealth index was calculated using PCA based on variables from the 2017 Indonesia Demographic and Health Surveys, including housing amenities, household assets and transportation ownership.

Pregnancy-related characteristics included: (i) unplanned pregnancy, (ii) number of antenatal care (ANC) visits and (iii) consumption of Fe and folic acid supplements. Unplanned pregnancy was categorised as ‘Yes’ if the current pregnancy was unplanned or undesired and ‘No’ if it was planned and desired. The number of ANC visits was classified as adequate if the woman attended at least six visits during pregnancy and inadequate if fewer than six. The consumption of Fe and folic acid supplements was categorised as ‘Yes’ if the woman had taken the supplements in the past 30 days and ‘No’ otherwise.

### Maternal stress, depression and common mental disorders

Several psychometric instruments were employed in this study to assess common mental health disorders (CMD), stress, depression and the level of social support among pregnant women. These instruments are widely used and have been validated in Indonesia^(28,29)^. The SRQ is a twenty-item scale developed by the WHO to measure symptoms of CMD^(30)^. This screening test has been validated for use during pregnancy and is widely applied in various settings, including the Indonesian National Health Survey^(31)^. The tool measures both psychological symptoms (items 4, 6, 8 and 9–17), such as feeling unhappy, frightened, worthless and low-energy, as well as common physical/somatic symptoms associated with anxiety and depression (items 1–3, 5, 7 and 18–20), such as headaches, sleep problems, low appetite and poor digestion^(32)^. A higher score of SRQ denotes higher levels of symptoms, which was converted into a binary variable where 1 = equal or greater than six ‘yes’ answers and 0 = less than six ‘yes’ answers^(31)^.

The second instrument, the GMPS, also known as Perceived Stress Scale, was used to determine maternal stress during the second trimester of pregnancy. Each item has a rating on a five-point Likert scale, where 0 represents ‘never’ and 4 represents ‘very often.’ The scale is clustered into two subscales, which are the positive subscale (items nos. 4, 5, 6, 7, 9, 10 and 13) and the negative subscale (items nos. 1, 2, 3, 8, 11, 12 and 14). The negative subscale, which comprises negatively stated items, is intended to assess a lack of control and negative reactions (perceived distress). The positive subscale measures the ability of individuals to cope with current stressors (coping capacity)^(33)^. Seven positive items were reverse-coded (e.g. 0 = 4, 1 = 3, 2 = 2 and 3 = 1, 4 = 0). The total score was calculated by summing the scores of all fourteen items, with a higher score indicating higher perceived stress. Scores ranging from 0–18 were classified as low stress, 19–37 as moderate stress and 38–56 as high stress, based on the categorisation established by Higgins^(34)^. In this study, maternal stress was categorised as low stress (score 0–18) and moderate to high stress (score 19–56). Previous research reported that the Perceived Stress Scale demonstrated good internal consistency (Cronbach’s *α* > 0·70) and test-retest reliability (> 0·70) for measuring stress among pregnant women^(35)^.

The third instrument, the EPDS, is a validated self-reported instrument comprising ten items. Each item is rated on a four-point Likert scale ranging from 0 (‘No, not at all/never’) to 3 (‘Yes, most of the time’)^(36)^. The total EPDS score ranges from 0 to 30, with higher scores indicating greater severity of depressive symptoms. The instrument demonstrated strong reliability and validity, with a Cronbach’s *α* of 0·86 and an AUC of 0·73^(37)^. It has been widely used as a screening tool for antenatal depression. In this study, the prevalence of antenatal maternal depression during the second trimester was estimated using a cut-off score of 9 out of a maximum of 30. The scores were then dichotomised into a binary variable, where 1 indicated a score greater than 9 (indicating depression) and 0 indicated a score of 9 or less^(38)^.

### Social support

Social support of pregnant women was measured using the MSPSS. There are three subscales: (a) family: items 3, 4, 8, 11; (b) friends: items 6, 7, 9 and 12 and (c) significant other: items 1, 2, 5 and 10, each of which addresses a different source of support. Each item was scored on a seven-point Likert scale (1 = ‘very strongly disagree’ to 7 = ‘very strongly agree’)^(39)^. The overall score was calculated by summing all twelve items, and the complete score ranges from 12 to 84. The score was then divided by 12 and categorised into a binary variable: 0 = high support (scores > 5) and 1 = low to moderate support (scores ≤ 5)^(40)^. This instrument demonstrated excellent internal consistency, with a Cronbach’s *α* of 0·93^(41)^.

### Biomarkers

Maternal venous blood samples were collected by phlebotomists during the third trimester to assess Hb level and Fe status (measured by ferritin concentration). Since plasma ferritin levels can be affected by inflammatory processes, not accounting for this may result in inaccurate estimates of true Fe status^(42)^. Therefore, acute-phase proteins, i.e., C-reactive protein (CRP) and *α*-1-acid glycoprotein (AGP)^(43)^, were also measured to adjust ferritin levels accordingly. The ferritin adjustment was performed following the standard methods recommended by the WHO^(44)^.

Blood samples were transported from study sites to the laboratory of Universitas Mataram Hospital for processing and Hb measurement. Subsequent analyses of Fe status and inflammatory markers were conducted at the SEAMEO RECFON laboratory using the Q-Plex Human Micronutrient 7-Plex ELISA assay (Quansys Biosciences). Subclinical inflammation was defined by CRP > 5 mg/l and AGP > 1 g/l^(45)^. The cut-off for ferritin < 12 μg/l was used to determine iron deficiency (ID), and Hb < 11 g/dl to determine anaemia during the third trimester of pregnancy^(46)^. Serum ferritin levels were adjusted for inflammation using the BRINDA approach^(47)^. Iron deficiency anaemia was defined as having both an adjusted ferritin level < 12 μg/l and Hb < 11·0 g/dl^(46)^.

### Dietary assessment

Dietary data were collected from pregnant women during the second trimester using the 24-h dietary recall with a four-pass multistage interviewing technique^(47)^. The four-pass 24-hour dietary recall consisted of: (1) recalling and listing all foods and beverages consumed the previous day; (2) collecting details on ingredients, cooking methods, composite foods and any other relevant information; (3) estimating portion sizes using food weighing and/or a food photobook and (4) verifying the recalled foods for completeness^(48)^.

To estimate portion sizes, trained enumerators followed a strict protocol that involved either weighing equivalent amounts of foods consumed or using a food photobook to approximate the portion sizes reported by mothers for the previous day. This approach aimed to improve accuracy while differing from the weighed food record method, which involves real-time weighing of foods as they are consumed. Weighing is one of several portion size estimation methods used in 24-h dietary recalls. Other portion size estimation methods included asking respondents to estimate portion of food samples, using food photographs and household measurement units^(49)^.

Enumerators brought food samples such as rice, noodles and bread or used foods available in the household. Respondents were asked to estimate the amounts of food and beverage consumed, after which the enumerators weighed the actual foods using a Tanita digital scale (model KD-160, precision ±1 g; Tanita Corporation, www.tanita.com). The food photobook, adapted from the 2014 Indonesian ‘Total Diet Study’ and modified to include locally typical foods, displayed gram weight equivalents for portion sizes alongside common kitchen utensils (e.g. tablespoon, cup and bowl) for reference^(50)^. These methods were employed to minimise bias from over- or under-reporting.

Minimum dietary diversity for women (MDD-W) was calculated using the dietary data. The ten MDD-W food groups, including (1) starchy staples (e.g. grains, white roots, tubers and plantations); (2) pulses (beans and peas); (3) nuts and seeds; (4) milk and dairy products; (5) meat, fish and poultry; (6) eggs; (7) dark green leafy vegetables; (8) vitamin A-rich fruits and vegetables; (9) other vegetables and (10) other fruits. Women received a score of 1 if they consumed at least 15 grams of food from each food group; otherwise, they received a score of 0. The dietary diversity score was calculated by adding all food group values, which were then converted into the binary MDD-W, where 0 = inadequate (< 5 food groups per day) or 1 = adequate (≥ 5 food groups per day)^(51)^.

Dietary pattern analysis was based on the original ten food groups defined by the FAO MDD-W. To identify different dietary patterns potentially associated with mental health outcomes, a more detailed classification was applied. Categories previously grouped together, such as meat, fish and poultry, were separated into three distinct groups. Additional food groups were included: (i) oils and fats, (ii) sweet foods, (iii) sugar-sweetened beverages, (iv) condiments, herbs and spices and (v) other beverages^(51)^, resulting in a total of eighteen food groups for analysis.

### Anthropometry measurement

Mid-upper arm circumference (MUAC) was used as a proxy to determine maternal nutritional status. MUAC was measured by trained enumerators, on the upper left arm, using a flexible and non-stretchable tape (SECA 201), positioned at the marked midpoint between the olecranon and acromion processes and measured to the nearest 0·1 cm^(52)^. Throughout the study period, all anthropometric instruments were calibrated regularly in accordance with the user’s manual. MUAC tape calibration was performed using a 30 cm stainless-steel ruler to ensure measurement accuracy and consistency. Chronic energy deficiency (CED) was defined as MUAC < 23·5 cm, while values > 23·5 cm were classified as normal nutritional status^(17)^.

### Ethical approval

The Action Against Stunting Hub Indonesia birth cohort study was conducted in accordance with the ethical principles outlined in the Declaration of Helsinki (2013). Ethical approval was obtained from the Institutional Ethics Committee of the London School of Hygiene and Tropical Medicine (Reference: 17915/RR/17513) and the Health Research Ethics Committee of the Faculty of Medicine, Universitas Indonesia–Cipto Mangunkusumo Hospital (FKUI-RSCM) (Reference Number: ND-914/UN2.F1/ETIK/PPM.00.02/2021). All participants provided written informed consent prior to enrollment. For those unable to sign, a witnessed thumbprint was obtained. Only pregnant women aged 18 years or older were eligible for recruitment, ensuring that minors were not included and that parental or guardian consent was not required.

Prior to data collection, trained enumerators clearly explained the study’s objectives and procedures. Informed consent was obtained from participants who agreed to take part. Participants were assured of the confidentiality and anonymity of their data, which were coded and used exclusively for research purposes. They were also informed that participation was entirely voluntary and that they could withdraw from the study at any time without any consequence.

### Data analyses

Descriptive results for categorical variables, such as socio-economic and maternal characteristics, were presented as frequencies with corresponding percentages. Dietary patterns were derived from eighteen food groups using PCA, with varimax rotation applied to achieve orthogonal (uncorrelated) factors. Food groups with absolute factor loadings ≥ 0·20 were considered to have strong associations with a given pattern and were used to define and label each dietary pattern^(53)^. The number of retained factors was determined based on eigenvalues (> 1·0), scree plot inspection and interpretability. The suitability of the dietary data for factor analysis was assessed using the Kaiser–Meyer–Olkin measure and Bartlett’s test of sphericity. The Kaiser–Meyer–Olkin value was 0·536, and Bartlett’s test yielded a *P* value < 0·001, indicating that the data were appropriate for factor analysis (acceptable criteria: Kaiser–Meyer–Olkin > 0·50 and *P* < 0·05). Five major dietary patterns with eigenvalues > 1·0 were identified, collectively explaining 19 % of the total variance in dietary intake. Adherence to each dietary pattern was categorised into quartiles: quartile 1 (low intake), quartile 2 (low-medium), quartile 3 (medium-high) and quartile 4 (high intake). For multivariable analysis, Q1 and Q2 were grouped as low adherence, while Q3 and Q4 were categorised as high adherence.

The PCA was also used to estimate the wealth index of study participants. The analysis used variables similar to those included in the 2017 Indonesia Demographic and Health Surveys^(54)^, such as housing quality (materials used for floors, walls and roofs), source of drinking water, type of cooking fuel, type of toilet facility, ownership of durable assets (e.g. television, refrigerator, bicycle, motorcycle and car) and other wealth-related indicators (e.g. ownership of a house, land or livestock). These variables served as explanatory inputs for the PCA. The overall wealth index score was categorised by tertile for analysis, where 1 = lowest wealth and 3 = highest wealth.

The *χ*^2^ test was used to assess associations between categorical variables. Multiple logistic regression analyses were conducted to identify factors associated with maternal stress, depression and common mental disorders. Variables with a *P* value < 0·20 in bivariable analysis were retained for inclusion in the multivariable models^(55)^. To avoid multicollinearity, highly intercorrelated variables were not included together; instead, variables with strong associations with the outcomes and/or those recognised as underlying risk factors for maternal mental health were prioritised. Statistical significance was set at *P* < 0·05. All analyses were performed using Stata version 16·0 (StataCorp).

## Results

Among the 724 pregnant women who met the eligibility criteria twenty-two declined to participate, resulting in a final sample size of 702 women (participation rate of 96.9 %). Complete data for the second trimester were obtained from all 702 participants. By the third trimester, the sample size decreased to 659 due to moving residence, miscarriage, molar pregnancy, maternal death or withdrawal from the study. Baseline characteristics of pregnant women, including nutritional status, socio-demographic and pregnancy-related characteristics, are presented in Table 1. The mean age of women was 28 years. Over half had completed secondary education (8–12 years), and the majority were housewives. Most fathers had attained secondary education (50·2 %) and were employed (98·3 %). More than 60 % of households consisted of 3–5 family members. None of the pregnant women were active smokers; however, a large majority (79·8 %) were exposed to secondhand tobacco smoke. Some pregnancies were unplanned, and nearly one-fifth (19·6 %) had fewer than six ANC visits. Additionally, 16·9 % did not consume Fe-folic acid supplements during pregnancy. Almost half (48·6 %) of the participants reported not receiving government assistance in the past 12 months. Approximately one-quarter of the women had inadequate dietary diversity, as defined by failing to achieve the MDD-W of consuming fewer than five of the ten food groups per day.

**Table 1.** Baseline characteristics of pregnant women in rural areas of East Lombok, Indonesia Table 1 long description.

Variable*	*n*	%
Maternal mental health		
Perceived stress, at T2 (score > 18)	606	86·3
Depression, at T2 (score > 9)	186	26·5
Common mental disorders, at T3 (score < 6)	196	29·7
Socio-economic and maternal characteristics		
Maternal age (years)		
< 25	213	30·4
25–29	207	29·6
≥ 30	280	40·0
Pregnant women’s education level (years of education)		
Primary (< 8 years)	167	23·8
Secondary (8–12 years)	463	65·9
Higher (> 12 years)	72	10·3
Pregnant women’s (housewives)	521	74·2
Father’s education level (years of education)		
Primary (< 8 years)	232	36·9
Secondary (8–12 years)	316	50·2
Higher (> 12 years)	81	12·9
Fathers’ employed	618	98·3
Family size		
1–2	185	26·3
3–5	423	60·3
≥ 6	94	13·4
Exposed to tobacco smoke (passive smokers)	560	79·8
Unplanned pregnancy	96	14·7
Insufficient ANC visits (< 6)	132	19·6
Did not consume iron-folic acid tablets	100	16·9
Did not receive government assistance (cash and/or food)	341	48·6
Inadequate dietary diversity at T2 (< 5 food groups/day)	169	24·4
Low to moderate perceived support, at T2 (score < 5)	244	34·8
Maternal nutritional status (anthropometry and biochemical)		
CED at T2	156	22·5
CED at T3	96	14·7
Anemia, at T3	270	41·4
ID, at T3^†^	392	60·1
ID, at T3^‡^	282	43·3
IDA, at T3	204	31·3
Low-grade inflammation (elevated CRP), T3	113	17·3
Chronic inflammation (elevated AGP), T3	22	3·4

T2, Trimester 2; T3, Trimester 3; ANC, Antenatal care; CED, Chronic energy deficiency; ID, Iron deficiency; ID, Iron deficiency anemia.

Cut-off for CED: MUAC < 23·5 cm; Anemia at T3: Hb < 11 g/dl; ID: ferritin < 15 mcg/l^†^ or ferritin < 12 mcg/l^‡^; IDA: anemia and ferritin < 12 mcg/l; Low-grade (systemic) inflammation: CRP > 5 mg/l; Chronic inflammation: AGP > 1 g/l.

* Number of subjects for each variable, T2:702, T3:659.

Antenatal stress was reported by a majority of pregnant women, with 86·3 % perceiving elevated stress levels. Depression was identified in 26·5 % of women during the second trimester (T2), while 29·7 % experienced CMD in the third trimester (T3). Regarding social support, approximately 34·8 % of participants perceived low to moderate levels during pregnancy (T2). The proportion of women with CED was 22·5 % and 14·7 % at T2 and T3, respectively. The prevalence of anaemia was high (> 40 %), and one-third of the pregnant women had iron deficiency anaemia.

Table 2 presents the dietary pattern of pregnant women along with their corresponding factor loadings. Five major dietary patterns were identified including Pattern 1: animal source foods, Pattern 2: traditional (less diverse), Pattern 3: less healthy (high sugar, high fat), Pattern 4: Healthy and diverse and Pattern 5: unhealthy beverages (high consumption of sugar-sweetened beverages).

**Table 2.** Dietary patterns of pregnant women and factor loadings using principal component analysis Table 2 long description.

Food group	Pattern 1	Pattern 2	Pattern 3	Pattern 4	Pattern 5
	Animal source foods	Traditional (less diverse)	Less healthy (high sugar, high fat)	Healthy, diverse	Unhealthy beverages (SSB)
Grains and cereals				0·579	
Roots and tubers				0·502	
Legumes		0·485			
Nuts and seeds			0·517		
Milk and its products					−0·262
Red meat	0·631				
Fish and seafood	0·666				
Poultry					0·659
Eggs				0·352	
Vitamin A-rich vegetables		0·514			
Vitamin A-rich fruits			0·571		
Other vegetables				0·298	
Other fruits				0·367	
Oils and fats			0·288		
Sweet foods			0·471		
Sugar-sweetened beverages (SSB)					0·522
Condiments, herbs, spices		0·507			
Other beverages					

Items with factor loadings ≥ |0·2| are presented in the table.

The first dietary pattern was characterised by a high intake of animal-source foods, including fish (e.g. skipjack tuna and freshwater fish), seafood and beef. The second pattern reflected a predominantly plant-based diet, marked by high consumption of vitamin A-rich vegetables (e.g. spinach, water spinach, moringa leaves, cassava leaves, carrots), legumes (e.g. tempeh, tofu, pigeon peas, soybeans) and a variety of condiments, herbs and spices (e.g. garlic, shallots, onions and chili peppers). The third pattern was distinguished by a high intake of vitamin A-rich fruits (e.g. mangoes, papayas), nuts and seeds (e.g. peanuts), as well as sweet foods (e.g. foods or dishes made with white or palm sugar, jelly, wafers) and fats/oils. This included fried foods and spicy dishes prepared with coconut milk, grated coconut or peanut-based sauces, which are commonly consumed as part of the traditional diet in East Lombok. The fourth pattern represented a healthy and diverse diet, comprising grains and cereals (e.g. rice, bread, cakes, tapioca crackers), tubers and roots (e.g. cassava, sweet potatoes), eggs (e.g. chicken and quail eggs), a variety of other vegetables (e.g. tomatoes, yard-long beans, eggplants, bean sprouts) and other fruits (e.g. dates, oranges, apples and bananas). The fifth pattern was characterised by high consumption of unhealthy beverages (e.g. iced drinks with sweetened condensed milk, coffee drinks, artificially flavored juices), high intake of poultry (particularly fried chicken) and low consumption of milk and dairy products, including formula milk intended for pregnant women.

Table 3 presents the results of multivariable logistic regression analyses examining the associations between socio-demographic, pregnancy-related and dietary factors with stress, depression and CMD among pregnant women in East Lombok, Indonesia. After adjusting for covariates, low adherence to dietary Pattern 4 (healthy and diverse) was significantly associated with increased odds of antenatal stress at T2 (aOR 1·59, 95 % CI 1·01, 2·52). In addition, maternal undernutrition or chronic energy deficiency (CED, aOR 1·73, 95 % CI 0·92, 3·24) and low to moderate support (aOR 1·62, 95 % CI 0·98, 2·68) demonstrated marginal associations with antenatal stress.

**Table 3.** Determinants of maternal depression, stress and common mental disorders in pregnancy Table 3 long description.

Variable	Perceived stress at T2						Depression at T2						Common mental disorders at T3					
	cOR	95·5 % CI	*P*-value	aOR	95·5 % CI	*P*-value	cOR	95·5 % CI	*P*-value	aOR	95·5 % CI	*P*-value	cOR	95·5 % CI	*P*-value	aOR	95·5 % CI	*P*-value
Maternal age (years)																		
< 25	1·09	0·66, 1·80	0·722	0·89	0·51, 1·54	0·667	**1·71**	**1·13, 2·60**	**0·005**	1·43	0·85· 2·38	0·174	0·98	0·65, 1·47	0·905	–		
25–29	1·65	0·95, 2·87	0·076	1·42	0·80, 2·53	0·236	**1·80**	**1·19, 2·71**	**0·012**	1·43	0·91, 2·24	0·121	1·14	0·76, 1·69	0·534			
≥ 30	Ref.			Ref.			Ref.			Ref.			Ref.					
Mother’s education level																		
Primary	Ref.			–			Ref.			Ref.			Ref.			Ref.		
Secondary	0·96	0·57, 1·62	0·888				**1·92**	**1·23, 3·00**	**0·004**	**1·65**	**1·01, 2·71**	**0·047**	1·48	0·98, 2·24	0·061	1·14	0·69, 1·88	0·608
Higher	0·84	0·38, 1·84	0·666				**2·38**	**1·26, 4·48**	**0·007**	**2·27**	**1·14, 4·49**	**0·019**	0·88	0·45, 1·74	0·712	0·86	0·37, 1·98	0·717
Mother’s employment status																		
Employed	Ref.			–			Ref.			–			Ref.			–		
Unemployed	1·22	0·76, 1·96	0·415				1·16	0·80, 1·70	0·430				0·89	0·60, 1·31	0·562			
Father’s education level																		
Primary	Ref.			–			Ref.			–			Ref.			Ref.		
Secondary	0·79	0·48, 1·29	0·344				1·25	0·85, 1·85	0·261				**1·67**	**1·12, 2·47**	**0·011**	1·46	0·93, 2·30	0·101
Higher	1·36	0·59, 3·09	0·470				1·31	0·74, 2·31	0·358				1·14	0·63, 2·08	0·663	1·20	0·57, 2·51	0·630
Father’s employment status																		
Employed	N/A (omitted)			–			Ref.			–			Ref.			–		
Unemployed							1·64	0·47, 5·66	0·437				1·40	0·41, 4·86	0·593			
Family size																		
1–2	Ref.			–			Ref.			Ref.			Ref.			–		
3–4	1·17	0·72, 1·91	0·533				**0·67**	**0·46, 0·98**	**0·040**	0·90	0·57, 1·43	0·664	0·85	0·58, 1·25	0·404			
≥ 5	1·22	0·59, 2·52	0·594				0·82	0·47, 1·41	0·468	1·07	0·58, 1·97	0·819	0·88	0·51, 1·54	0·664			
Tobacco smoke exposure																		
No	Ref.			–			Ref.			–			Ref.			–		
Yes	0·97	0·56, 1·66	0·909				1·18	0·77, 1·82	0·441				0·95	0·63, 1·44	0·808			
Wealth status																		
High wealth	Ref.			Ref.			Ref.			Ref.			Ref.			–		
Medium wealth	1·04	0·59, 1·83	0·886	1·01	0·58, 1·76	0·973	1·46	0·96, 2·22	0·073	1·04	0·67, 1·61	0·860	1·18	0·79, 1·77	0·412			
Low wealth	0·64	0·38, 1·08	0·092	0·91	0·51, 1·61	0·750	1·35	0·88, 2·05	0·166	1·23	0·79, 1·94	0·361	0·88	0·58, 1·33	0·532			
Receive government assistance																		
Yes	Ref.			–			Ref.			–			Ref.			Ref.		
No	1·19	0·77, 1·84	0·425				0·96	0·69, 1·34	0·817				1·37	0·98, 1·92	0·065	**1·48**	**1·01, 2·17**	**0·046**
Social support (T2)																		
High support	Ref.			Ref.			Ref.			Ref.			Ref.			Ref.		
Low to moderate support	1·51	0·94, 2·44	0·091	1·62	0·98, 2·68	0·061	**1·83**	**1·30, 2·58**	**0·001**	**1·72**	**1·20, 2·46**	**0·003**	1·33	0·94, 1·89	0·103	1·45	0·98, 2·15	0·063
ANC visits																		
Sufficient	Ref.			Ref.			Ref.			–			Ref.			–		
Insufficient	0·66	0·40, 1·09	0·107	0·62	0·36, 1·05	0·073	0·94	0·60, 1·45	0·77				1·25	0·83, 1·88	0·295			
Dietary diversity (T2)																		
Adequate	Ref.			–			Ref.			–			Ref.			–		
Inadequate	0·88	0·53, 1·44	0·598				1·06	0·72, 1·57	0·755				0·95	0·65, 1·41	0·812			
Dietary pattern 1: Animal source foods (ASF)																		
Q3–Q4 (High)	Ref.			–			Ref.			–			Ref.			Ref.		
Q1–Q2 (Low)	0·78	0·50, 1·21	0·268				0·86	0·61, 1·21	0·388				1·37	0·97, 1·91	0·07	1·20	0·82, 1·75	0·340
Dietary pattern 2: Traditional (less diverse)																		
Q3–Q4 (High)	1·35	0·87, 2·10	0·174	1·39	0·88, 2·20	0·159	1·10	0·71, 1·71	0·657	–			1·02	0·73, 1·42	0·930	–		
Q1–Q2 (Low)	Ref.			Ref.			Ref.						Ref.					
Dietary pattern 3: Less healthy (high sugar, high fat)																		
Q3–Q4 (High)	0·95	0·61, 1·46	0·801	–			1·40	1·00, 1·96	0·052	**1·47**	**1·03, 2·10**	**0·036**	0·85	0·61, 1·19	0·355	–		
Q1–Q2 (Low)	Ref.						Ref.			Ref.			Ref.					
Dietary pattern 4: Healthy and diverse																		
Q3–Q4 (High)	Ref.			Ref.			Ref.			–			Ref.			Ref.		
Q1–Q2 (Low)	**1·73**	**1·11, 2·71**	**0·015**	**1·59**	**1·01, 2·52**	**0·045**	1·06	0·76, 1·49	0·73				**1·51**	**1·08, 2·12**	**0·017**	**1·60**	**1·10, 2·34**	**0·015**
Dietary pattern 5: Unhealthy (SSB)																		
Q3–Q4 (High)	1·10	0·71, 1·71	0·657	–			1·09	0·78, 1·53	0·604	–			**0·64**	**0·46, 0·90**	**0·011**	0·75	0·51, 1·09	0·131
Q1–Q2 (Low)	Ref.						Ref.						Ref.					
Nutritional status (T2)																		
Normal	Ref.			Ref.			Ref.			Ref.			Ref.			–		
CED	1·62	0·91, 2·91	0·103	1·73	0·92, 3·24	0·088	**1·58**	**1·07, 2·32**	**0·021**	1·24	0·81, 1·88	0·321	1·15	0·77, 1·70	0·502			
Nutritional status (T3)																		
Normal													Ref.			Ref.		
CED													1·54	0·98, 2·42	0·063	1·20	0·71, 2·01	0·499
Anemia status T3																		
Normal													Ref.			–		
Anaemic													1·24	0·88, 1·75	0·223			
Iron deficiency T3																		
Normal													Ref.			Ref.		
ID													**1·43**	**1·02, 2·01**	**0·040**	**1·61**	**1·11, 2·35**	**0·012**
IDA T3																		
Normal													Ref.			–		
IDA													1·35	0·94, 1·94	0·099			

ANC, Antenatal care; CED, Chronic energy deficiency; cOR, crude odd ratio; aOR, adjusted odd ratio; T2, trimester 2; T3, trimester 3; Ref., reference; Q, quartiles; SSB, sugar-sweetened beverages; ID, iron deficiency, IDA, iron deficiency anaemia.

The bold ones are the variables that have p-value <0.05 that indicates a significant association.

* Statistical analysis was performed using logistic regression. Variables with a *P* value < 0·2 in the bivariable analysis were included in the multivariable model.

Bivariable analysis showed that the likelihood of antenatal depression during the second trimester (T2) was higher among women with CED (cOR 1·58, 95 % CI 1·07, 2·32), young age i.e. 18–25 years (cOR 1·71, 95 % CI 1·13, 2·60), women with higher educational attainment (cOR 2·38, 95 % CI 1·26, 4·48) and those reporting low to moderate levels of social support (cOR 1·83, 95 % CI 1·30, 2·58). Conversely, women living in households with 3–4 members (cOR 0·67, 95 % CI 0·46, 0·98) were less likely to experience depression. In the multivariable analysis, significant determinants of antenatal depression included higher maternal education (aOR 2·27, 95 % CI 1·14, 4·49), low to moderate social support (aOR 1·72, 95 % CI 1·20, 2·46) and high adherence to dietary Pattern 3, characterised as a less healthy diet high in sugar and fat, which was associated with increased odds of depression (aOR 1·47, 95 % CI 1·03, 2·10).

Multivariable logistic regression analysis showed that pregnant women with iron deficiency (aOR 1·61, 95 % CI 1·11, 2·35), those who did not receive government assistance (aOR 1·48, 95 % CI 1·01, 2·17) and those with low adherence to a healthy and diverse dietary pattern (Pattern 4) (aOR 1·60, 95 % CI 1·10, 2·34) were at significantly higher risk of CMD during the third trimester.

## Discussion

In this study, the majority of pregnant women (over 86 %) reported perceived stress, and approximately 30 % experienced antenatal depression and CMD. Nutritional factors associated with increased risks of stress and CMD were low adherence to a healthy and diverse dietary pattern, as well as iron deficiency (for CMD only). In contrast, antenatal depression was more strongly associated with non-nutritional factors, including low social support and higher maternal education.

Our findings are consistent with those of a study conducted during the COVID-19 pandemic, which reported stress and depression in 70 % and 31 % of pregnant women, respectively^(56)^. These prevalence rates are notably higher than those observed under non-pandemic conditions, as shown in cross-sectional studies from Indonesia and China, where the proportions of maternal stress and depression were 55·6 % and 29·6 %, respectively^(57,58)^. The lockdowns, physical distancing and other containment measures implemented during the pandemic have been associated with an increased risk of psychological distress among women, due to psychosocial stressors such as fear of illness, social isolation and economic uncertainty^(59)^. Moreover, disruptions in healthcare systems may have limited pregnant women’s access to ANC, resulting in inadequate counseling on health and nutrition, as well as reduced emotional support, factors that are crucial for both maternal mental well-being and the health of the baby^(60)^.

Limited access to ANC exacerbates existing health challenges, such as iron deficiency during pregnancy, which remains a significant public health issue in low- and middle-income countries^(61,62)^. Iron deficiency has been shown to significantly influence the development and management of depression^(63)^. Although no association was found between iron deficiency and depression in our study, it was identified as a risk factor for CMDin the third trimester. Pregnant women with iron deficiency had a 1·6-fold increased risk of developing CMD, suggesting its involvement in physiological processes affecting mental health^(64)^. Previous studies have indicated that low Fe levels may cause hypomyelination of neurons, impairing the production and release of neurotransmitters such as dopamine and serotonin, which are essential in the pathophysiology of depression^(65,66)^. Iron deficiency, the leading cause of anaemia in low- and middle-income countries, especially during pregnancy, necessitates the inclusion of standard health education messages during ANC visits that encourage pregnant women to consume Fe-rich foods and take Fe-containing supplements.

In the bivariable analysis, women suffering from CED were 1·58 times more likely to experience depression during pregnancy. This finding is consistent with a cross-sectional study conducted in Kenya, which reported a positive association between poor nutritional status (MUAC < 23 cm) and maternal depression^(67)^. These findings suggest that inadequate diet quality during pregnancy contributes to poor nutritional status, which in turn increases the risk of maternal depression.

A diverse diet is essential for supporting both physical and mental health. A systematic review by Głabska *et al.* found that a high intake of fruits and vegetables, particularly subgroups such as berries, citrus and green leafy vegetables, may reduce psychological distress and protect against depressive symptoms^(68)^. Additionally, study shows that consuming a nutritionally adequate diet is associated with improved mental health outcomes^(69)^. Thus, low adherence to a healthy and diverse diet, comprising ASF (particularly eggs), grains/tubers, fruits and vegetables, was associated with an increased risk of CMD and stress in this study. Conversely, pregnant women with high adherence to a diet high in sugar and fat were more likely to experience depression (aOR 1·47).

Our findings align with a recent systematic review by Khaled *et al.*, which reported a significant association between stress and unhealthy dietary patterns, characterised by high intake of fat, salt and fast food, alongside low intake of fish, vegetables, fruits and unsaturated fats^(70)^. Similarly, a study conducted among Japanese women found that low intake of eggs and vegetables had a significantly higher likelihood of experiencing depression^(71)^. Egg yolks and dark green leafy vegetables are rich sources of methyl donor nutrients, including folate, riboflavin, vitamin B_6_, vitamin B_12_, methionine, betaine and choline^(72,73)^. These nutrients are essential for brain function and neurotransmitter regulation (particularly dopamine, serotonin and norepinephrine). Deficiencies in methyl donors can disrupt one-carbon metabolism and methylation processes, thereby contributing to the development of depressive disorders^(67,74)^. These findings underscore the importance of promoting dietary diversity during pregnancy as part of nutrition education messages to support maternal mental health.

The relationship between dietary intake and maternal mental health may be reciprocal: poor mental health can lead to a poor diet, including low dietary diversity, while a good diet can promote more positive emotions and improved mental well-being^(68)^. During pregnancy, it is recommended to consume at least 2–3 portions of ASF and 4–5 portions of fruits and vegetables daily, consistent with the Indonesian balanced dietary guidelines^(75)^.

In our study, pregnant women who did not receive government assistance (whether through cash transfers or food aid) had a 1·48 times higher risk of developing CMD during the third trimester. Since 2007, the Indonesian government has implemented the conditional cash transfer program, *Program Keluarga Harapan* (PKH), which supports impoverished families with pregnant women and/or young children. Additionally, the *Bantuan Pangan Non Tunai* (BPNT) provides food aid, including rice, eggs, poultry, meat and vegetables. These programmes play a crucial role in protecting women from lower socio-economic backgrounds, particularly during the pandemic, by improving their access to diverse and nutritious diets^(76)^.

We found that the risk of antenatal depression increased approximately 2·4-fold among women with higher education (college level or above). This finding aligns with previous studies conducted before the pandemic, which reported that women with higher education were more likely to experience antenatal depression compared with those with lower education levels^(58,77)^. Di Florio *et al.* further observed that women with higher education levels are more prone to self-harm, excessive crying and irrational worries^(77)^. A plausible explanation is that women with higher education tend to have greater access to various media sources (e.g. television, social media and newspapers), exposing them to extensive health information about health knowledge and making them more attentive to their mental health^(58)^.

Social support emerged as a significant factor influencing mental health in this study. Low levels of social support were strongly associated with an increased risk of antenatal depression, consistent with findings from a previous cohort study in China^(1)^. Adequate social support has been shown to buffer against stress by attenuating physiological stress responses, including reductions in sympathetic nervous system activation, hypothalamic–pituitary–adrenal axis activity and inflammatory reactivity^(78)^. This study was conducted during the COVID-19 pandemic, a period during which many women may have experienced heightened isolation, particularly those lacking support from partners, family members or other relatives. Mothers of young children who received assistance with household tasks and newborn care had a significantly lower risk of depression compared with those who experienced disruptions in support system during the pandemic^(79)^.

### Strengths and limitations

One of the key strengths of this study is its longitudinal design, as part of the Action Against Stunting Hub Indonesia project, which allowed for comprehensive data collection from pregnant women over time. Direct interaction with participants through door-to-door visits helped minimise attrition and maintain data quality, while adhering to social distancing protocols during the COVID-19 pandemic. The cohort achieved a high participation rate (96.9%) and adhered to standardised operational procedures throughout the data collection process, resulting in a robust dataset and enhancing the reliability of our findings. To our knowledge, this is the first study in Indonesia to assess maternal mental health using three validated mental health scales among pregnant women. The use of standardised, validated instruments further strengthens the reliability and validity of the data collected. However, several limitations should be acknowledged. Due to the observational nature of the study, causal inferences between the determinants of maternal stress and depression cannot be established. Additionally, since data collection took place during the COVID-19 pandemic, the findings may not fully reflect experiences during non-pandemic conditions.

### Conclusions

Our findings indicate that a substantial proportion of pregnant women experienced perceived stress, depression and CMD and underscore the importance of preventing iron deficiency and promoting healthy, diverse diets as strategies to reduce the risk of psychological distress during pregnancy. Nutrition-specific interventions, including dietary diversification, food-based recommendations and Fe-folate supplementation, are essential during this critical period and may have positive effects on both the mental and physical well-being of pregnant women. In addition, our results highlight the critical role of social support in mitigating the risk of depression and mental disorders during pregnancy, which can have adverse implications for child development^(59)^. Integrated approaches that combine nutrition-specific interventions, government assistance for economically disadvantaged households and enhanced social support from family, friends and the community are recommended to improve maternal mental health during pregnancy.

## Table 1 Long description

The table presents baseline characteristics of pregnant women in rural areas of East Lombok, Indonesia, focusing on maternal mental health, socio-economic factors, and nutritional status. It includes data on perceived stress, depression, common mental disorders, maternal age, education levels, family size, exposure to tobacco smoke, and various socio-economic indicators. The table also covers maternal nutritional status, including anemia, iron deficiency, and inflammation levels. Key trends include high percentages of perceived stress and exposure to tobacco smoke, as well as varying levels of education and employment among the participants. The table consists of multiple rows and columns, detailing specific variables and their respective percentages and counts.

Navigate back to Table 1.

## Table 2 Long description

The table presents dietary patterns of pregnant women and their corresponding factor loadings. It includes five major dietary patterns: animal source foods, traditional (less diverse), less healthy (high sugar, high fat), healthy and diverse, and unhealthy beverages (high consumption of sugar-sweetened beverages). The table has 16 rows and 5 columns. The columns are labeled Pattern 1, Pattern 2, Pattern 3, Pattern 4, and Pattern 5. The rows are labeled with different food groups such as grains and cereals, roots and tubers, legumes, nuts and seeds, milk and its products, red meat, fish and seafood, poultry, eggs, vitamin A-rich vegetables, vitamin A-rich fruits, other vegetables, other fruits, oils and fats, sweet foods, sugar-sweetened beverages (SSB), condiments, herbs, spices, and other beverages. Each cell contains a numerical value representing the factor loading for that food group within the respective dietary pattern.

Navigate back to Table 2.

## Table 3 Long description

The table presents the results of multivariable logistic regression analyses examining the associations between socio-demographic, pregnancy-related, and dietary factors with stress, depression, and common mental disorders among pregnant women in East Lombok, Indonesia. The table includes variables such as maternal age, mother's education level, mother's employment status, father's education level, father's employment status, family size, tobacco smoke exposure, wealth status, receipt of government assistance, social support, ANC visits, dietary diversity, dietary pattern, nutritional status, and anemia status. Notable findings include the significant association between low adherence to dietary Pattern 4 (healthy and diverse) and increased odds of antenatal stress at T2, with an adjusted odds ratio (aOR) of 1.59 and a 95% confidence interval (CI) of 1.01 to 2.52. Additionally, maternal undernutrition or chronic energy deficiency (CED) and low to moderate support demonstrated marginal associations with antenatal stress, with aORs of 1.73 and 1.62, respectively.

Navigate back to Table 3.
